# The effect of basketball matches on salivary markers: a systematic review

**DOI:** 10.5114/biolsport.2022.107481

**Published:** 2021-10-25

**Authors:** Paulius Kamarauskas, Daniele Conte

**Affiliations:** 1 Institute of Sport Science and Innovations, Lithuanian Sports University, Kaunas, Lithuania

**Keywords:** Hormonal response, Testosterone, Cortisol, Physiological demand, Psychological demand

## Abstract

The aim of this paper was to synthesize the findings on salivary marker responses to the different basketball match typologies. An electronic database search of articles published until October 2020 was performed in PubMed, SPORTDiscus, Scopus and Web of Science. Studies were then screened using pre-defined selection criteria and a subsequent assessment of methodological quality was conducted. Articles matching the selection criteria and methodological quality were included in the systematic review. The electronic database search produced 696 articles. After removing 505 duplicates, 191 articles were included for screening. Screening led to 10 articles that met the inclusion criteria. The main findings revealed that playing a basketball match induced a highly stressful condition reflected by increased post-match cortisol levels regardless of season phase (i.e. regular vs. semi-final vs. final matches), match outcome (i.e. winning vs. losing matches) and location (i.e. home vs. away). Different results were found for testosterone, which showed inconsistent outcomes when measured before and after matches. However, an effect of match location on testosterone levels was observed, with higher concentrations before home matches compared to away matches. Finally, playing basketball matches led to an increase in levels of alpha-amylase, a decrease in interleukin-21 and no changes in immunoglobulin A, total protein and brain-derived-neurotrophic factor. The current results provide a detailed description of salivary markers changes in response to different basketball matches, which can help practitioners to have a better understanding of the basketball performance profile.

## INTRODUCTION

Saliva collection and analysis is considered as an established tool for assessment of the physiological responses in sports training and matches [1]. This method has been extensively used since it can provide a useful, non-invasive alternative to the collection of serum and plasma. Indeed, saliva can be collected rapidly and frequently, and it can be performed in the sports field without any medical training [1]. Additionally, the use of saliva samples for hormonal analysis was recommended since it reduces the risk of possible infections compared with blood analysis, and has lower overall cost and higher acceptability by athletes [2–4].

Salivary markers during official matches in team sports and specifically in basketball have been extensively studied since they can provide useful information about the physiological and psychological profile of the athletes before and after these events [5]. In basketball, official matches have been shown to be characterized by high physical [6] and physiological [6–8] demands, leading to increased physical and psychophysiological stress [6, 9, 10] and disturbing the balance between anabolic and catabolic processes [11]. Therefore, the assessment of salivary markers evaluating these processes before or after official basketball matches has been considered fundamental [12]. Cortisol (C) is among the most studied markers to assess the acute responses during official basketball matches [12–19]. Specifically, C is secreted from the adrenal cortex via the hypothalamic–pituitary–adrenal (HPA) axis and is the main hormone responsible for the catabolic process as it reduces protein synthesis, increases protein degradation and inhibits the inflammatory process and immunity [1]. Therefore, C is the main marker identifying athletes’ stress responses in matches, which is one of the most stressful events in sport settings [20]. Testosterone (T) is the primary androgen steroid hormone and its secretion is regulated by the hypothalamic-pituitary-gonadal axis [1]. T is the main hormone responsible for the anabolic process, including muscle growth, since it increases protein synthesis and consequently muscle strength-related performance [21]. Therefore, T was mainly used in sport settings to monitor the anabolic processes during resistance training [21, 22]. Alternatively, from a psychological standpoint, T has been shown to be linked to the concept of dominance, territory and aggression, and was measured during matches as a marker indicating athletes’ arousal and aggression levels when playing at home vs. away venues and when winning or losing official basketball matches [12, 23].

The current body of literature indicates that several markers have been studied to assess the psychological profile of basketball players according to different match conditions such as different final outcomes [15], match venues [12], phases of the season [14], difficulties of the match [13, 24], comparing responses to simulated and official matches [18, 19] and effects of matches and exercise on levels of hormonal response [16]. However, to the best of our knowledge, there has been no systematic review of changes in salivary markers according to different basketball match conditions, which could indicate a comprehensive understanding of the basketball match demands from a physiological and psychological standpoint. Therefore, the aim of this systematic review is to synthesize the findings about salivary markers’ responses to the different basketball match typologies.

## MATERIALS AND METHODS

### Literature search strategy

An electronic database search for the articles published online or in print prior to October 2020 was performed in four electronic databases: PubMed, SPORTDiscus, Scopus and Web of Science. The search strategy presented in Table 1 consisted of three search variables (Salivary markers AND Type of activity AND Basketball) used in all possible combinations for the identification of relevant publications. Identified original peer-reviewed articles published in English were considered as relevant search outcomes while literature reviews, conference proceedings and other types of publications were excluded.

**TABLE 1:** Search strategy used to locate relevant research articles.

	Variable	Search terms
1.	Salivary markers	(“hormonal response*” OR “salivary cortisol” OR “salivary testosterone” OR “salivary immunoglobulin A” OR “salivary marker*” OR “endocrinology”)
2.	Type of activity	(“game*” OR “match*”)
3.	Basketball	(“basketball”)

Salivary markers AND type of activity AND basketball “1 AND 2 AND 3”	

### Selection criteria

The selection criteria of this systematic review were created and used with no restrictions for study population, interventions, comparisons, outcomes and study designs (PICOS), following recommendations [25] and search strategies used in other systematic reviews [6, 26, 27]. During the screening process, publications investigating the effect of basketball matches (i.e. official, friendly, or simulated) on changes in salivary markers were included in the review. The article screening process was performed following the guidelines of Preferred Reporting Items for Systematic Reviews and Meta-Analyses (PRISMA) [28].

After exclusion of duplicate records, the abstracts of all identified articles were screened independently against the pre-defined selection criteria by two authors (PK and DC). The full-text version screening process was then performed in the same structure for all included articles. Additionally, the reference list of all included articles was screened by two authors (PK and DC), to identify any relevant articles that were not found during the database search. The considered salivary markers in this systematic review were C, T, IgA, inter-leukin-1ß (IL-1ß), interleukin-21 (IL-21), alpha-amylase (AA), brain-derived neurotrophic factor (BDNF), and total protein (TP).

### Assessment of methodological quality

The modified version of the Downs and Black checklist for assessment of methodological quality of randomised and non-randomised healthcare interventions [29] was used. The Downs and Black checklist was proved as a valid method [29] and has been previously used in systematic reviews to assess methodological quality [30–32]. Following the recommendations that the number of items used for the assessment of methodological quality can be adjusted to the scope of the systematic review [30–32], the checklist for this review was adapted for non-interventional and for interventional study designs, consisting of the 12 and 13 most relevant items, respectively (Table 2). During the assessment of methodological quality, each included article was independently evaluated by two authors (PK and DC) and each item was assessed as 1 = “Yes”, or 0 = “No/unable to determine”. The scores for each of the 12 or 13 items were summed to provide the total quality score.

**TABLE 2:** Questions of the modified Downs and Black checklist used for the assessment of methodological quality of the included articles.

Question	
No.	Reporting
**1**	Is the hypothesis/aim/objective of the study clearly described?
**2**	Are the main outcomes to be measured clearly described in the Introduction or Methods section?
**3**	Are the characteristics of the patients/subjects included in the study clearly described?
**4**	Are the main findings of the study clearly described?
**5**	Does the study provide estimates of the random variability in the data for the main outcomes?
**6**	Have actual probability values been reported (e.g. 0.035 rather than < 0.05) for the main outcomes except when the probability value is less than 0.001?
**External validity**	
**7**	Were the subjects asked to participate in the study representative of the entire population from which they were recruited?
**8**	Were those subjects who were prepared to participate representative of the entire population from which they were recruited?
**Internal validity**	
**9**	If any of the results of the study were based on “data dredging”, was this made clear?
**10**	In trials and cohort studies, do the analyses adjust for different lengths of follow-up of patients, or in case control studies, is the time period between the intervention and outcome the same for cases and controls?
**11**	Were the statistical tests used to assess the main outcomes appropriate?
**12**	Were the main outcome measures used accurate (valid and reliable)?
**13**	Was compliance with the intervention/s reliable? *(Just for interventional studies)*

### Data extraction and analysis

To identify and extract representative data from all the included articles, publications were analysed by the lead author (PK). Nonnumerically presented or unprovided data were identified as “not reported”. During the identification and extraction process, the following data were extracted (if presented):

– Characteristics of participants: sample size, playing level, sex, age, stature and body mass;
– Research methodology: salivary markers, use of saliva flow rate stimulation, use of mouth rinse before collection, dietary restrictions due to saliva collection, collection type (i.e. swabbing, spitting), manufacturer of reagents used for analysis;
– Methodological outcome measures: phase of the season, duration of monitoring period, type of activities monitored, frequency of saliva sample collection, salivary markers analysed and variability in results of analysis of salivary markers;

Study results: outcomes of saliva analysis (i.e. differences, statistical significance, effect sizes and interpretation).

Where possible, participants’ characteristics are reported as mean ± standard deviation (SD) and the type of methodology used to collect saliva samples is presented in Table 3.

**TABLE 3:** Types of methodology used to collect saliva samples in the included articles.

Study	Salivary markers	Stimulated Yes / No	Mouth rinse Yes / No	Dietary restriction time	Collection type	Manufacturer of reagents
Arruda et al. 2018 [14]	Cortisol	No	No	90 min	Spitting	Salimetrics
	Testosterone					
	Alpha-amylase					
	IL-1b cytokine					

Arruda et al. 2014 [12]	Cortisol	No	No	90 min	Spitting	Salimetrics
	Testosterone					

Gonzalez-Bono et al. 1999 [15]	Cortisol	Yes	No	n/a	Spitting	ICN (T) OD (C)
	Testosterone					

Moreira et al. 2018 [16]	Cortisol	No	No	90 min	Spitting	Salimetrics (C)
	Brain-derived neurotrophic factor					Abnova (BDNF)

Moreira et al. 2013 [17]	Cortisol	No	No	120 min	Spitting	
	Immunoglobulin A					Salimetrics (C; IgA)
	Interleukin-21					eBio (IL-21)
	Total protein					Pierce (TP)

Moreira et al. 2012(a) [18]	Cortisol	No	No	120 min	Spitting	ALPCO
	Immunoglobulin A					

Moreira et al. 2012(b) [19]	Cortisol	No	No	120 min	Spitting	DSL

Arruda et al. 2019 [24]	Testosterone	No	No	90 min	Spitting	Salimetrics

Arruda et al. 2017 [13]	Cortisol	No	No	90 min	Spitting	Salimetrics
	Testosterone					

Gonzalez-Bono et al. 2000 [33]	Testosterone	Yes	No	n/a	Spitting	ICN

*Note.* n/a – not available, not provided in article; Salimetrics – Salimetrics LLC, Carlsbad, CA, USA; ICN – ICN Biomedicals, Costa Mesa, CA, USA; OD – Orion Diagnostica, Espoo, Finland; Abnova – Abnova Corporation, Taiwan; eBio – eBioscience, San Diego, CA, USA; Pierce – Pierce Biotechnology, Rockford, Illinois, USA; ALPCO – ALPCO diagnostics, Salem, MA, USA; DSL – Diagnostic Systems Laboratories, INC, Webster, TX, USA; C – cortisol; T – testosterone; IgA – immunoglobulin A; AA – alpha-amylase; IL-1b – IL-1b cytokine; BDNF – brain-derived neurotrophic factor; IL-21 – interleukin-21.

## RESULTS

### Search findings and study selection

**FIG. 1:**
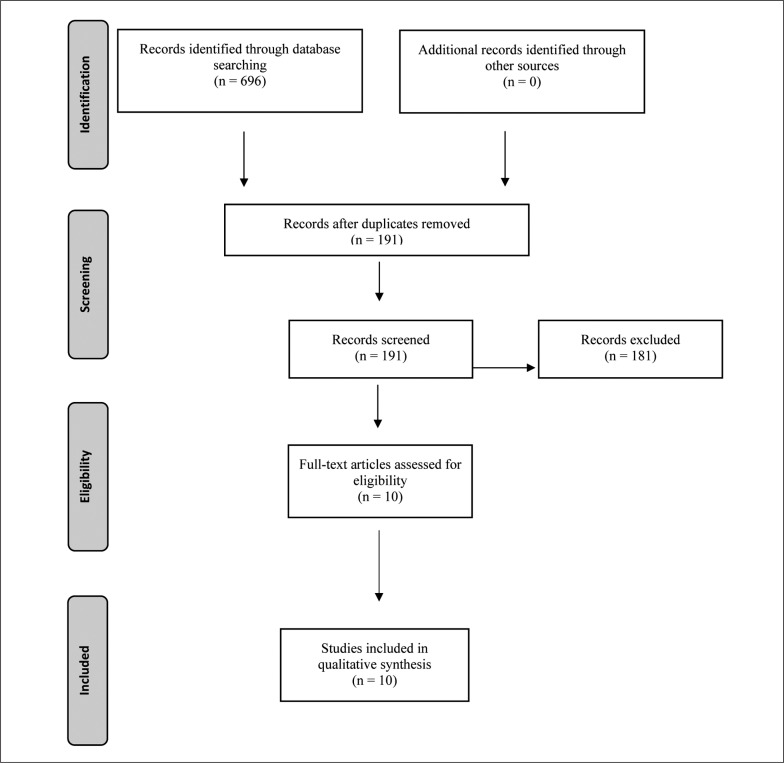
Preferred Reporting Items for Systematic Reviews and Meta-Analysis (PRISMA) flow diagram of search strategy.

A total of 696 articles were found across the electronic databases (PubMed = 152, SPORTDiscus = 88, Scopus = 167, Web of Science = 289) and, after removing 505 duplicate records, 191 records were included for a further analysis of eligibility. After screening titles and abstracts, a further 181 articles were removed before the full-text screening procedure with the remaining ten (n = 10) articles passing the final full-text screening procedure matching all the selection and evaluation criteria. The full results of the search are presented in Figure 1.

### Methodological quality

The results of methodological quality evaluation for each included article are presented in Table 4. The total scores for non-interventional studies (maximum possible score = 12) ranged from 7 to 10, while the two intervention studies both recorded a score of 9 (maximum possible score = 13). Similarly to other systematic reviews that used the Downs and Black checklist [26, 30–32], no articles were excluded based on the results of methodological quality evaluation.

**TABLE 4:** Results of methodological quality assessment for included articles.

Study	Downs and Black checklist question number													TOTAL
	Reporting						External validity			Internal validity-bias				
	1	2	3	4	5	6	7	8	9	10	11	12	13	
Arruda et al. 2018 [14]	1	1	1	0	0	1	0	1	1	0	1	1	T	8
Arruda et al. 2014 [12]	1	1	1	1	1	0	0	1	1	0	1	1	T	9
Gonzalez-Bono et al. 1999 [15]	1	1	1	0	0	1	0	0	1	1	1	1	1	9
Moreira et al. 2018 [16]	1	1	1	0	0	0	0	1	1	1	1	1	1	9
Moreira et al. 2013 [17]	1	1	1	0	0	0	0	1	1	0	1	1	T	7
Moreira et al. 2012(a) [18]	1	1	1	1	1	0	0	1	1	1	1	1	T	10
Moreira et al. 2012(b) [19]	1	1	1	0	0	1	0	1	1	1	1	1	T	9
Arruda et al. 2019 [24]	1	1	1	0	0	1	1	1	1	0	1	1	T	9
Arruda et al. 2017 [13]	1	1	1	0	0	0	0	1	1	0	1	1	T	7
Gonzalez-Bono et al. 2000 [33]	1	1	1	1	1	1	0	0	1	0	1	1	T	9

*Note.* 1 = Yes; 0 = No/Unable to determine; T – non-interventional study.

### Participant characteristics

Participants’ characteristics are presented in Table 5. Included studies investigated samples with different sizes, ranging from 10 to 25 participants included for the final analysis. Salivary markers across basketball matches were studied only in male players across all the included articles. Participants from included articles were competing at the elite basketball level in youth (n = 7) and senior (n = 3) age categories.

**TABLE 5:** Characteristics of the participants in included articles.

Study	Sample size (N)	Level	Sex	Age (years)	Stature (cm)	Body mass (kg)
	Final [Initial]			(mean ± SD)	(mean ± SD)	(mean ± SD)
Arruda et al. 2018 [14]	14 [18]	Elite	Male			
	U16 (N = 7)			U16: 15.1 ± 0.3	U16: 190.3 ± 9.1	U16: 90.4 ± 15.5
	U17 (N = 7)			U17: 16.5 ± 0.5	U17: 191.5 ± 7.2	U17: 89.7 ± 18.9

Arruda et al. 2014 [12]	18 [24]	Elite	Male	17.8 ± 0.4*	190 ± 10*	87 ± 8.5*

Gonzalez-Bono et al. 1999 [15]	16 [21]	Elite	Male	W: 23.63 ± 1.22 #	W: 195.78 ± 1.95 #	W: 93.04 ± 3.84 #
	Winners (W)					
	(N = 8) Losers			L: 22.86 ± 1.82 #	L: 195.41 ± 2.6 #	L: 94.59 ± 3.49 #
	(L) (N = 8)					

Moreira et al. 2018 [16]	24 [33]	Sedentary	Male			
	Sedentary adults	adults				
	(S) (N = 12)	Elite		S: 23.0 ± 4.2*	S: n/a	S: n/a B:
	Basketball players	basketball		B: 18.6 ± 0.5*	B: 192.7 ± 7*	88.9 ± 14.5*
	(B) (N = 12)	players				

Moreira et al. 2013 [17]	20	Elite	Male	18.8 ± 0.4	192 ± 10	87 ± 8

Moreira et al. 2012(a) [18]	10	Elite	Male	19 ± 0.6	193 ± 6	87 ± 7

Moreira et al. 2012(b) [19]	10	Elite	Male	26.4 ± 3.8	196 ± 10	100 ± 14

Arruda et al. 2019 [24]	25 [33]	Elite	Male			
	U15 (N = 8)			U15: 14.1 ± 0.3	U15: 186.6 ± 6.9	U15: 78.3 ± 12.2
	U16 (N = 8)			U16: 15.2 ± 0.4	U16: 191.0 ± 8.1	U16: 88.9 ± 13.8
	U17 (N = 9)			U17: 16.5 ± 0.5	U17: 191.5 ± 7.2	U17: 89.7 ± 18.9

Arruda et al. 2017 [13]	12	Elite	Male	18.6 ± 0.5	192 ± 7	88.9 ± 14.5

Gonzalez-Bono et al. 2000 [33]	17	Elite	Male	T1: 21.56 ± 1.16 #	T1: 194.84 ± 2.10 #	T1 : 92.99 ± 3.85 #
	Team 1 (T1)					
	(N = 9) Team			T2: 22.0 ± 1.70 #	T2: 193.83 ± 2.76 #	T2: 92.50 ± 3.67 #
	2 (T2) (N = 8)					

*Note.* n/a – not provided;

* - average data reported for initial sample size; SD – standard deviation.

# – data reported as mean ± SEM (standard error of the mean).

### Outcome measures

Outcome measures of the included articles are presented in Table 6. Different markers were used, with C and T being the most studied markers: C (n = 8), T (n = 6), IgA (n = 2), AA (n = 1), BDNF (n = 1) IL-1ß (n = 1), IL-21 (n = 1), TP (n = 1). In all included articles, saliva samples were collected from before and after investigated matches and in some cases comparing basketball match results with specifically designed exercises, training or laboratory sessions, depending on the purpose of each study. In the identified studies, we also considered whether the coefficient of variation (CV) values were reported for intra- and inter-assay, which are typical analyses used to verify the reliability of measurements (Table 6). The results revealed that CVs were reported in: i) 8 (intra-assay; range: 3.4%–7.0%) and 1 (inter-assay; value: < 5.0%) articles out of 8 assessing C levels; ii) 6 (intra-assay; range: 3.2%–5.0%) and 2 (inter-assay; value: < 5.0%) articles out of 6 assessing T levels; and iii) 2 (intra-assay; range: 6.0%–7.0%) and 0 (inter-assay) articles out of 2 assessing IgA levels. For other salivary markers, CVs were reported only for intra-assay with values of 2.6% for AA, 6.7% for IL-1ß, 8.0% for BDNF and 3.2% for IL-21, while for TP CVs were not reported.

**TABLE 6:** Methodological outcome measures of included articles

Study	Duration	Type of activity	Frequency of saliva collection	Salivary markers	Coefficient of variation for the assays (%)
Arruda et al. 2018 [14]	4 matches	Two winning playoff final matches and two winning regular season matches.	Pre- to post-match.	C	C = 4.4 intra
				T	T = 4.6 intra
				AA	AA = 2.6 intra
				IL-1b	IL-1b = 6.7 intra

Arruda et al. 2014 [12]	2 matches	Two teams played against each other twice, playing at home and away facilities.	Pre- to post-match.	C	C = 3.7 intra
				T	T = 3.2 intra

Gonzalez-Bono et al. 1999 [15]	9 months	Experimental laboratory session in August, December and April and experimental match in December for two teams.	Pre- to post-match and during 3 laboratory sessions.	C	< 5.0 intra & inter
				T	

Moreira et al. 2018 [16]	6 weeks	Basketball players participated in 3 official matches. Sedentary group visited the laboratory 4 times to complete an experimental protocol at 120% of HRV_TH_ for 30 minutes.	Pre- to post-match and experimental exercise.	C	C = 3.6 intra
				BDNF	BDNF = 8.0 intra

Moreira et al. 2013 [17]	1 match	Two teams, 1^st^ and 2^nd^ place in the Brazilian State Basketball Championship played regular season matches against each other.	Pre- to post-match.	C	C = 4.8 intra
				IgA	IgA = 6.0 intra
				IL-21	IL-21 = 3.2 intra
				TP	TP = n/a

Moreira et al. 2012(a) [18]	15 weeks	5 investigated basketball matches: 2 official and 3 training matches.	Pre- to post-match.	C	< 7.0 intra
				IgA	

Moreira et al. 2012(b) [19]	4 weeks	4 investigated basketball matches: 2 official and 2 simulated matches.	Pre- to post-match.	C	4.8 intra

Arruda et al. 2019 [24]	6 winning matches	3 winning semi-final and 3 winning final matches for U15, U16 and U17 teams.	Pre- to post-match.	T	4.6 intra

Arruda et al. 2017 [13]	9 weeks	Experimental training session and 3 official matches against different level of opponents.	Pre- to post-match and training session	C	C = 3.4 intra
				T	T = 4.2 intra

Gonzalez-Bono et al. 2000 [33]	2 matches	Two matches against different level opponents.	Pre- to post-match.	T	< 5.0 intra & inter

*Note.* n/a – not available and not provided in article; HRV_TH_ – heart rate variability threshold; C – cortisol; T – testosterone; IgA – immunoglobulin A; AA – alpha-amylase; IL-1b – IL-1b cytokine; BDNF – brain-derived neurotrophic factor; IL-21 – interleukin-21.

### Salivary marker responses to basketball matches

Two studies examined changes of salivary markers following basketball matches [17, 33] with eight additional studies considering different match outcomes (winning vs. losing) [15], match locations (home vs. away) [12], part of the season (regular vs. final) [14], difficulty of the match (final vs. semi-final and different level of opponents) [13, 24], and in comparison with simulated matches [18, 19] and with sedentary individuals performing exercise [16] (Table 7).

**TABLE 7:** Effect of basketball matches on salivary marker levels

Study	Marker	Measures	Concentration (mean ± SD)	Changes
Arruda et al. 2018 [14]	C	Regular vs. final matches	Not provided	p = 0.36
		Pre- to post-match measures		p < 0.001
		Effect of interaction		p = 0.09

	T	Regular vs. final matches	Not provided	p = 0.28
		Pre- to post-match measures		p < 0.001
		Effect of interaction		p = 0.35

	AA	Pre- to post-regular and final matches	Not provided (Increased)	p < 0.001
		Effect of interaction	Not provided	p = 0.58
		Effect of condition		p = 0.67

	IL-1ß	Pre- to post-regular and final matches	Not provided	p = 0.95
		Effect of interaction		p = 0.75
		Effect of condition		p = 0.57

Arruda et al. 2014 [12]	C	Home vs. away matches	Pre-home Pre: 19.5 ± 5.2 nmol/l	p > 0.05
			Post-home: 31.4 ± 7.6 nmol/l	
		Pre- to post-matches (home and away)	Pre-away Pre: 19.1 ± 5.7 nmol/l	p < 0.005
			Post-away: 28.5 ± 9.5 nmol/l	

	T	Home vs. away matches	Pre-home: 701 ± 146 nmol/l	p > 0.05
		Pre-match: home vs. away	Pre-away: 531 ± 153 nmol/l	p < 0.001
		Post-match: home vs. away	Post-home: 944 ± 243 nmol/l	p > 0.05
		Changes from pre- to post-match values	Post-away: 770 ± 257 nmol/l	p < 0.005

Gonzalez-Bono et al. 1999 [15]	C	Winners vs. losers (effect of match	Winners: 3.07 ± 1.31 nmol/l	p > 0.05
		outcome)	Losers: 1.59 ± 1.15 nmol/l	
		Pre- to post-match	Not provided	p < 0.02

	T	Winners vs. losers (effect of result)	Not provided	p > 0.05
		Pre- to post-match	W increase: 0.013 ± 0.04 nmol/l	p > 0.05
	Winners (W)			
	Losers (L)		L decrease: –0.031 ± 1.31 nmol/l	

Moreira et al. 2018 [16]	C	Group effect (Basketball players vs. Sedentary people)	Not provided	p < 0.001
		Time effect (Basketball players vs. Sedentary people)		p < 0.001
		Interaction (Time x Group)		p < 0.001

	BDNF	Group effect (Basketball players vs. Sedentary people)	Not provided (Higher in basketball players)	p < 0.001
		Time effect (Basketball players vs. Sedentary people)		p > 0.05
		Interaction (Time x Group)		p > 0.05

Moreira et al. 2013 [17]	C	Pre- to post-official basketball match	Not provided (Increased during the match)	p < 0.05
	IgA		Not provided	p > 0.05
	Secretion rate IgA		Not provided	p > 0.05
	IL-21		Not provided (Decreased)	p < 0.05
	TP		Pre: 1.7 ± 0.8 mg/ml	p = 0.7
			Post: 1.9 ± 0.9 mg/ml	

Moreira et al. 2012(a) [18]	C	Pre- to post-official basketball matches	Pre: 6.1 ± 0.8 nmol/l	p < 0.05
			Post: 12.7 ± 2.2 nmol/l	
		Pre- to post-training basketball matches	Pre: 4.2 ± 0.7 nmol/l	p > 0.05
			Post: 4.4 ± 1.0 nmol/l	
		Pre-match levels for official (OM) and training (TM) matches	OM: Pre: 6.1 ± 0.8 nmol/l	p > 0.05
			TM: 4.2 ± 0.7 nmol/l	
		Post-match levels for official (OM) and training (TM) matches	OM: 12.7 ± 2.2 nmol/l	p < 0.05
			TM: 4.4 ± 1.0 nmol/L	

	IgA	Pre- to post-official (OM) and training (TM) matches	Pre-OM: 457 ± 68 ug/ml	p > 0.05
			Post-OM: 552 ± 59 ug/ml	
			Pre-TM: 494 ± 99 ug/ml	
			Post-TM: 635 ± 137 ug/ml	

	Secretion rate IgA	Pre- to post-official (OM) and training (TM) matches	Pre-OM: 132 ± 30 ug/min	p > 0.05
			Post-OM: 156 ± 26 ug/min	
			Pre-TM: 118 ± 22 ug/min	
			Post-TM: 145 ± 31 ug/min	

Moreira et al. 2012(b) [19]	C	Pre- to post-simulated matches (SM)	Not provided	p > 0.05
		Pre- to post-official matches (OM)		p < 0.01
		Comparison of pre-OM and pre-SM		p < 0.03
		Comparison of post-OM and post-SM		p < 0.01

Arruda et al. 2019 [24]	T	Pre- to post-semi-final and final matches	Not provided (Increased)	p < 0.001
		Semi-final vs. final match	Not provided	p = 0.20
		Interaction between conditions		p = 0.93

Arruda et al. 2017 [13]	C	Time effect (pre- to post-matches)	Not provided (Increased)	p < 0.0001
		Condition effect (TS, EM, MM, HM)	Not provided (Increased)	p < 0.0001
		Pre- to post-all conditions (TS, EM, MM, HM)	Not provided (Increased)	p < 0.05
		Comparison of pre-match concentrations (TS vs. EM vs. MM. vs. HM)	Not provided (Higher in HM than in TS, EM and MM)	p < 0.05
		Comparison of post-match concentrations (TS vs. EM vs. MM. vs. HM)	Not provided (Higher in HM than in TS and EM)	p < 0.05

	T	Pre-match (EM, MM, HM) vs. pre-control (TS) concentrations	Not provided (Higher before all matches than before control session)	p = 0.008
		Comparison of pre-match concentrations	Not provided	p > 0.05

Gonzalez-Bono et al. 2000 [33]	T	Pre-match: T1 vs. T2	T1 pre-match: 0.078 ± 0.017 nmol/l *	p > 0.05
		Pre- to post-match for T1		p < 0.058
			T1 post-match: 0.116 ± 0.025 nmol/l *	
		Pre- to post-match for T2	T2 pre-match: 0.087 ± 0.009 nmol/l *	p < 0.97
			T2 post-match: 0.087 ± 0.016 nmol/l *	

*Note.*

* – data are reported as mean ± SEM (standard error of the mean);

C – cortisol; T – testosterone; AA – alpha-amylase; IL-1ß – interleukin-1ß; BDNF – brain-derived-neurotrophic factor; IgA – immunoglobulin A; IL-21 – interleukin-21; OM – official match; TM – training match; SM – simulated match; TS – training session; EM – easy match; MM – medium match; HM – hard match; T1 – team 1; T2 – team 2.

Previous one-match studies evaluating pre- to post-match differences revealed no statistically significant differences (p > 0.05) in T levels in professional basketball players [33] and no differences in TP and IgA in under-19 youth male basketball players [17]. However, significantly different (p < 0.05) C and IL-21 levels were observed comparing pre- to post-match values with an increase of C levels and a decrease in IL-21 [17] in under-19 youth male players. The analysis of the effect of match outcome showed no significant differences between winning and losing teams in C and T levels with C levels significantly increasing from pre- to post-match values for both teams and T showing no significant difference [15].

Considering match location, a previous investigation [12] revealed higher (p < 0.05) pre-match T values in home compared to away venues, while no significant difference was found for C concentrations. Moreover, both salivary markers increased (p = 0.005) from pre- to post-match values for both home and away matches [12]. The analysis of different salivary markers (C, T, AA, IL-1ß) showed no differences (p > 0.05) for matches played in different phases of the season (regular vs. final phase), with a statistically significant increase (p < 0.001) of C, T and AA levels from pre- to post-match values in both regular and final phase matches [14].

The analysis of the effect of matches with different difficulty levels showed no statistically significant differences (p > 0.05) for T concentrations in pre-match values when comparing final and semi-final matches [24] and easy, medium and hard matches [13]. Additionally, T values similarly increased (p < 0.001) from pre- to post-match values when playing both semi-final and final matches [24]. In contrast, C levels were found to be affected by different levels of opponents (easy, medium and hard matches) and time (pre- to post-matches) [13]. Indeed, higher (p < 0.05) C values were reported before hard matches compared to easy matches, and when considering post-match values, higher C concentrations were observed in hard matches compared to easy and medium matches [13].

When assessing differences in salivary marker concentrations between official and simulated matches in elite male senior and youth players, a higher (p < 0.05) pre-match C concentration was obser ved for official matches compared to simulated matches [18, 19]. Additionally, the C concentration significantly increased following official matches (p < 0.05), while no differences were found in simulated matches, with these results indicating a significantly higher C concentration after official compared to after simulated matches [18, 19]. However, playing in an official or simulated match did not affect the IgA values in elite youth basketball players, with IgA concentration remaining similar before and after matches in both conditions [18].

Finally, in a unique study the differences in C and BDNF concentrations in young adult male sedentary people involved in 30 minutes of constant load exercise at 120% of their heart-rate variability threshold in comparison with elite under-19 male basketball players involved in two official basketball matches were investigated [16]. Increased (p < 0.05) C levels from pre- to post-match values were observed in basketball players, while no changes were found for the sedentary group [16]. Moreover, post-activity and post-match C levels were higher in basketball players compared to sedentary people [16]. A higher (p < 0.05) resting and post-exercise BDNF concentration was found in basketball players compared to the sedentary group, with no changes found for both groups from pre- to post-exercise values [16].

## DISCUSSION

The aim of this systematic review was to synthesize findings on salivary marker responses to the different basketball match typologies. The main salivary markers included in the reviewed articles were C, T and IgA and are discussed in separate sections.

### Reliability of results

Higher reliability of results indicates high precision of measurements with CV as one of the most useful calculations adopted for this analysis [34]. Specifically, for the assessment of salivary markers, acceptable reliability is considered when CV for intra- and inter-assays is lower than 10% [35]. The results of this systematic review indicate that the reliability values were reported in all included articles with coefficient of variation values < 10% (Table 6).

### Cortisol

Regardless of match typology (i.e. regular, semi-final, final), outcome (i.e. winning, losing), location (i.e. home, away), and level of opponent (i.e. easy, medium, hard), all eight reviewed articles reported an increase in C levels following an official match [12–19]. Furthermore, higher C levels were observed following official matches compared to simulated matches in elite [19] and youth [18] male basketball players. An increase in C levels indicates that official matches possess a less controlled environment compared to simulated matches, generating higher stress levels, and leading to greater psychophysiological demands [20]. The reasons for higher stress might relate to the interactions with other players, changes in match circumstances, pressure from the coach or fans, self-efficacy, anxiety and psychological pressure to win [17, 36–38].

The level of opponents has also been indicated as a variable able to increase the C levels in elite male basketball players [13]. Indeed, higher pre-match C concentrations were found before hard matches compared to training sessions, easy matches and medium matches [13]. Moreover, C concentrations following hard matches were higher than following easy matches and training sessions [13]. These outcomes can be explained by greater perceived threat of failure and individual stress due to playing against a highly ranked team, resulting in higher activity of the hypothalamic-pituitary-adrenal axis, which increases the release of stress hormones [13, 14]. Additionally, playing in an official match has been demonstrated as a highly stressful condition regardless of season phase (i.e. regular vs. semi-final vs. final matches) [14, 24], match outcome (i.e. winning vs. losing) [15] and location [12], since no differences were found when comparing these variables in elite male senior and youth players.

### Testosterone

When considering changes in levels of T, previous research focused on the assessment of pre-match T levels according to contextual factors such as match location [12] and level of the opponents [13] in elite youth male basketball players. Higher T concentrations were found before home compared to away matches [12], while no differences were found when comparing pre-match values of matches against differently ranked opponents [13]. These findings suggest that players perceived higher self-confidence when playing at home [12, 23], while the level of opponents did not induce any changes in pre-match T levels, probably because social status provocation would be an essential characteristic in any match [13].

Conversely to C outcomes, an inconsistent response of T was found from pre- to post-match values for official matches [12, 14, 15, 24, 33]. Basketball matches elicited an increase in T with a similar trend found when comparing matches played in different phases of the season (i.e. regular, semi-final, final) [14, 24] and matches played in different locations (home vs. away) [12]. The increase of T levels following matches can be explained by psychological responses to challenging conditions, such as maintaining a high social status and overcoming threats of failure [13, 39]. However, dissimilar results were obtained in previous studies assessing the changes in T levels from pre- to post-match values when winning matches [33] or comparing concentrations of winning and losing teams [15], with no changes documented. However, it is worth mentioning that when considering absolute values, an increase in T levels was reported for winners and a decrease for losers [15]. This inconsistency in the results across the reviewed manuscripts calls for further research to determine a more precise response of T levels to basketball matches according to different contextual variables and in different age categories.

### Immunoglobulin A

An increase in training stress and C levels was previously suggested as associated with neuroendocrine control and elevation of IgA levels [40]. However, an increase in C concentration from before to after official matches in youth and senior elite male basketball players [17, 18] did not have an impact on IgA concentrations, with no changes observed. The unresponsiveness of IgA concentration during official basketball matches shows that an acute increase in C levels has no effect on regulation of IgA levels [17, 18]. Possibly, IgA concentrations might be considered a less useful salivary markers to assess players’ short-term responses since it has been suggested that immunological responses occur with a certain delay [41].

### Other salivary markers

Besides the analysis of C, T and IgA, three included articles investigated the responses of AA, IL-1ß, IL-21, TP and BDNF following basketball matches [14, 16, 17]. Salivary AA and IL-1ß were previously indicated as markers of stress and immune responses, respectively [42, 43]. A previous study assessing the changes in AA and IL-1ß following regular and final matches in elite youth players indicated different responses [14]. Indeed, an increase in AA concentration was found concurrently with an increase in C levels [14], suggesting the combination of these two markers as providing a more detailed interpretation about the activity of the sympathetic nervous system (SNS) [42], which strongly depends on the level of psychological stress [14, 44]. On the other hand, no changes for IL-1ß were found following regular and final matches [14]. Possibly, this outcome was due to the raised level of inflammatory cytokines to elicit greater stimulation of the hypothalamic-pituitary-adrenal axis to release C, which resulted in inhibition of inflammatory cytokine production, responsible for the release of IL-1ß [45]. This negative feedback loop between the immune system and the CNS has been indicated as critical in regulating inflammatory responses and maintaining players’ health status [14, 45, 46].

Another salivary marker which was suggested as an important cytokine for the acute response to infections is IL-21, which is responsible for rapid production of IgA [17]. Moreover, IL-21 was described as a cytokine having both pro-inflammatory and anti-inflammatory effects on IgA [47]. However, a decrease in IL-21 following a match in youth basketball players did not cause any changes in IgA levels, although an acute increase in stress levels was found [17]. Contrary to the suggestions of rapid IgA production, findings confirm a delayed immunological response and usefulness of IgA for assessment of short-term changes, since a decrease in IL-21 did not induce an acute occurrence in IgA levels [41].

When considering the other investigated salivary markers, Moreira et al. [17] assessed the pre- to post-match TP levels in youth male basketball players, finding no statistically significant changes. TP has been suggested as a marker representing the whole body hydration status, with its increase showing a loss of body fluids [48]. This mechanism can be explained by the increase in SNS activity in response to the heat stress during exercise, leading to the acute reduction of hydration and lower saliva flow rate, resulting in higher saliva osmolality and an increase in TP levels [48, 49]. Therefore, a constant level of TP reveals that youth players were able to maintain a similar level of hydration during the match, which was not affected by the increased stress levels [17, 48].

Another salivary marker which was found to be unaffected by a basketball match in elite male players and by high-intensity exercise in sedentary people is BDNF [16]. This salivary marker was concomitantly assessed with C levels, demonstrating an increase in C secretion corresponding to no changes in BDNF following a basketball match [16]. These results might be due to the protective role of BDNF from stress-induced functional changes in the hippocampus and amygdala, which are responsible for control of motivation, emotions, learning and memory [50]. In fact, the only difference found was higher BDNF levels in basketball players than in sedentary individuals [16]. This difference shows that, similarly to C levels, regardless of the level of fitness or adaptation to experiencing a physical load, an official match is a more demanding condition than high-intensity exercise [16]. The lack of changes in BDNF is possibly related to the type of analysis as salivary BDNF level might not reveal the actual changes of BDNF in brain and muscle, since plasma BDNF might possess a higher responsiveness [16, 45]. This is the only investigation in basketball including salivary BDNF; thus further research is required to reduce speculations.

Overall, we would suggest to basketball practitioners and sport scientists the use of salivary markers as a valuable monitoring tool during basketball matches to assess the psychophysiological profile of basketball players. Future research directions should involve the analysis of several basketball populations and particularly in female basketball athletes since no previous studies have investigated their salivary marker responses to official basketball matches. Moreover, we suggest the design of studies also including other workload measures (i.e. heart rate, rating of perceived exertion and microtechnology) to assess the performance profile of basketball matches.

## CONCLUSIONS

This review is the first to provide a systematic evaluation of the changes in salivary markers in response to different typologies of basketball matches. The reported findings show that playing a basketball match induced a highly stressful condition reflected by increased post-match C levels regardless of season phase (i.e. regular vs. semi-final vs. final matches), match outcome (i.e. winning vs. losing matches) and location (home vs. away). Different results were found for T levels, which showed inconsistent outcomes measuring pre- and post-match values. However, an effect of match location on T levels was found, with higher concentrations before home matches compared to away matches. Finally, playing basketball matches led to an increase in AA, a decrease in IL-21 and no changes in IgA, TP and BDNF levels.

## Funding

This research did not receive any specific grant from funding agencies in the public, commercial, or not-for-profit sectors.

## Conflict of interest

The authors declare that they have no conflict of interest with the content of this systematic review.

## Author contributions

Paulius Kamarauskas 50%; Daniele Conte 50%.
